# CH_3_NH_3_Pb_1−*x*_Eu_*x*_I_3_ mixed halide perovskite for hybrid solar cells: the impact of divalent europium doping on efficiency and stability†

**DOI:** 10.1039/c7ra12754e

**Published:** 2018-03-20

**Authors:** Xiaowei Wu, Hongwei Li, Kai Wang, Xiaowei Sun, Liduo Wang

**Affiliations:** National Engineering Research Center for Rare Earth Materials, General Research Institute for Nonferrous Metals, Grirem Advanced Materials Co., Ltd. Beijing 100088 China lihw0923@vip.126.com; Department of Electrical & Electronic Engineering, Southern University of Science and Technology of China Shenzhen 518055 China; Key Lab of Organic Optoelectronics and Molecular Engineering of Ministry of Education, Department of Chemistry, Tsinghua University Beijing 100084 China chldwang@mail.tsinghua.edu.cn

## Abstract

The crucial role of the impact of divalent europium doping in perovskite solar cells is investigated in this work. We selected europium (Eu^2+^, 117 pm) to replace lead (Pb^2+^, 119 pm) because their ion radii are really comparable. This appropriate substitution has shown great potential to achieve high stability and enhance the power conversion efficiency of the solar cells. Through adjusting the doping concentration of europium, the perovskite solar cells corresponding average efficiency greatly increased. Furthermore, compared with the CH_3_NH_3_PbI_3_ perovskite film, the attenuation of power conversion efficiency of europium doped perovskite film slowed down 4.7 times at room temperature. Therefore, we put forward a useful method for the optimization of organic–inorganic perovskite solar cells.

## Abstract 2

The crucial role of the impact of divalent europium doping in perovskite solar cells is investigated in this work.

## Graphical abstract

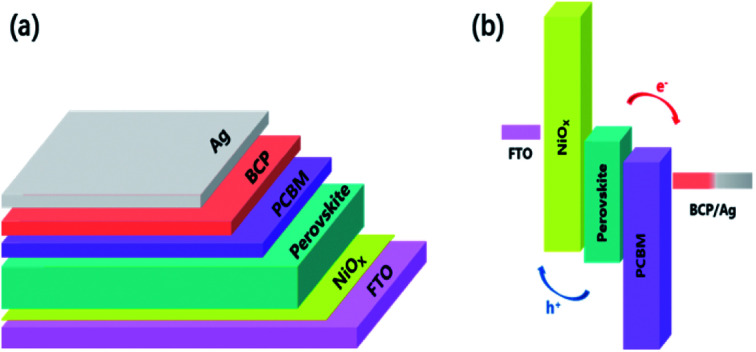

## Introduction

The recently arisen organic–inorganic halide hybrid perovskite, as a star in the family of multifunctional materials, has attracted extensive interest from both scientific and industrial communities. The materials are applied in many fields, such as photovoltaics,^1,2^ photodetectors,^3,4^ light-emitting diodes,^5^ lasers,^6,7^ and electrochromism.^8^ Organic–inorganic hybrid halide perovskite materials are commonly based on the ABX_3_ structure, where A is a monovalent cation (methylammonium (MA^+^), formamidinium (FA^+^), *etc.*), B is a divalent metal ion (Pb^2+^, Sn^2+^, Ge^2+^, Sr^2+^, Cd^2+^, Ca^2+^, *etc.*), and C is a monovalent anion (Cl^−^, Br^−^, I^−^, SCN^−^, *etc.*). This kind of material possesses several advantages such as low cost solution processability, large absorption coefficients, tunable band-gaps, high extinction coefficients, long carrier lifetimes, and high carrier mobility.^9–14^ In the past few years, the power conversion efficiency (PCE) of perovskite-based solar cells (PSCs) increased rapidly from 3.8% to 22.1% accompanied with the development of the large-area module processing techniques,^15–18^ which almost caught up with or even exceed the efficiency of crystalline silicon, CIGSe (copper–indium–gallium–selenide) and CdTe solar cells.^19–21^ In order to achieve the high performance and better stability of the solar cell, the perovskite thin film should be pinhole-free, grain boundary cracks-free, smooth, dense and well-defined crystalline.^22–28^ As a result, various methods were developed to improve these qualities of perovskite thin film, for example interfacial engineering, solvent engineering, additive-assisted preparation, *etc.*^29–34^

Doping of semiconductors, a process of intentional inserting impurities into an extremely pure crystal, is a key approach to manipulate optical and electrical properties of device.^35–39^ In general, new energy states within the band gap are introduced by doping, so as to facilitate the occurrence of low-energy electronic transitions.^35,40,41^ MAPbX_3_ are primarily doped by the elements isovalent with cations (Li^+^/Na^+^/Rb^+^), anions (I^−^/Cl^−^) or metal cations (Pb^2+^/M^2+^, M^2+^ = Sn^2+^, Sr^2+^, Cd^2+^, Zn^2+^ or Ca^2+^).^9,34,42–47^ In lead halide perovskites, MA and Pb cations are surrounded by 12 and 6 halide anions, respectively, which makes it to be a superb candidate to accommodate a variety of foreign ions with ionic radii comparable that of MA or Pb. However, this phenomenon limits the dopants' capacity, so as to change the majority charges from positive to negative or impact the concentration of the majority charge carriers.^39^

The previous work reported that the ions (Bi^3+^, Li^+^, Na^+^, K^+^, Rb^+^) are doped into the halide perovskites, just as a topotactic insertion.^38,39,41,42,48^ On one hand, the successfully incorporating dopants into perovskite crystals, and preserving the host lattice structures simultaneously, only cause tiny lattice distortion. On the other hand, these dopants oppose oxidation and reduction owing to their stability or resistance hence are chosen to be the positive additive.^39^ Nevertheless, there are, still many other elements not been explored.

Rare earth (RE) is a special down-conversion functional material. RE cation, as an activation center or dopants, has been widely used in automobile catalysts, petroleum refining catalysts, phosphors in color television and flat panel display, permanent magnets, rechargeable batteries for hybrid and electric vehicles, numerous medical devices.^43^ RE ions have the ability absorb UV light and re-emit visible light due to their specific 4f electronic structure.^44–47^ It is a kind of ideal candidate for extending the spectral response range of perovskite material to the UV region and enhancing the PSCs efficiency while reducing the UV degradation to improve the device stability.^48^ It was reported that in the dye sensitized solar cells (DSSCs), trivalent RE doping can reduce intra-gap trap states in anatase, which affects the electron diffusion length, and the low RE concentration in the lattice will affect the unit cell parameters in the structure. In the DSSCs, the doped Eu and Tb created an impurity energy level, giving rise to the absorbance spectra shift.^46^ Much research using RE to improve the PSCs performance of PSCs has been proposed.^49–51^ However, the influence of RE doping in the PSCs have not been reported yet.

Hence, based on the special properties of RE elements, we propose embedding Eu^2+^(117 pm), which is only slightly smaller than Pb^2+^(119 pm), into a photoactive perovskite phase.^52,53^ We introduced halide of rare earth EuI_2_ as a dopant embedded into MAPbI_3_. After doping, the absorbance of the thin film changed, since the Eu^2+^ has many different energy level transition orbits, the absorbance of the thin film was improved through doping bivalent RE ion (Eu^2+^), and the fill factor (FF) increased caused by reducing the PbI_2_ phase precipitate and enlarging the grain size. The PCE of the device with efficiency of 16.7%, was significantly promoted with 25% compared with the control sample. And the perovskite stability was enhanced slightly in air because of the disappearance of bad phase of PbI_2_.

## Experimental

### Materials synthesis

Methylammonium iodide (CH_3_NH_3_I) was synthesized and purified based on the method proposed by J. H. Im.^54^ All chemicals were used as received. Methylammonium iodide (CH_3_NH_3_I) was synthesized by mixing methylamine (CH_3_NH_2_) (27.8 mL, 0.273 mol, 40 wt% in methanol, Alfa Aesar) and hydroiodic acid (HI) (30 mL, 0.227 mol, 57 wt% in water, Alfa Aesar) in a 250 mL round-bottom flask, and stirring the mixture in an ice-water bath for 2 h. The yellowish raw product obtained by evaporating the solvent was recrystallized three times from a mixture of diethyl ether and ethanol. After filtration, the solid was collected in a dark container and dried at 60 °C in a vacuum oven overnight. Anhydrous EuI_2_ was synthesized and purified based on the method proposed by Chengpeng D.^55^ Anhydrous EuI_2_ was prepared by dissolving europium oxide (Eu_2_O_3_) and ammonium iodide (NH_4_I) into HI to form a transparent solution. We obtained a dense solid after we evaporated the solution. Then the solid was placed into a quartz tube for vacuum dehydration in a tube heating furnace, until it was completely dehydrated. After that, the dehydrated solid was sintered until the solid turned to transparent melt. Eventually, anhydrous EuI_2_ in bulk polycrystalline was obtained.

### Fabrication of solar cells

Devices were fabricated on fluorine-doped tin oxide (FTO) coated glass (Yingkou OPV Tech New Energy CO., LTD., OPV-FTO22-7). Initially, FTO was etched with 2 mol L^−1^ HCl solution and zinc metal powder. Substrates were then cleaned sequentially by soap solution (2 vol% Hellmanex™ detergent), deionized water, acetone, ethanol, isopropanol (IPA) and UV exposure. Nickel(ii) acetylacetonate was dissolved in ethanol (0.1 mol L^−1^) with adding 5.3 μL ethanolamine (38 wt%) into the solution. The solution was then stirred in a sealed glass vial in air overnight. Then the NiO solution was spin-coated onto the UV–ozone treated FTO substrate at 3000 rpm for 60 s and then annealed at 400 °C for 60 min in ambient.

The perovskite thin film was deposited by using a process similar to that described in a previous work.^47^ The MAPbI_3_ : *x*% EuI_2_ (*x* = 0, 0.02, 0.04, 0.06, 0.08, 0.1) precursor solution was prepared by dissolving CH_3_NH_3_I (1 mmol), PbI_2_ (1.05 mmol; Alfa Aesar, 99.9985%) and EuI_2_ (a corresponding mass fraction of PbI_2_) in *g*-butyrolactone (GBL)/dimethyl sulfoxide (DMSO) (7 : 3; 1 mL) with a total concentration of 1 M and stirring at 70 °C overnight. The perovskite thin films spread with 80 μL was spin coated onto the FTO/NiO substrate followed by a two-stage spin-coating process at 1000 rpm for 15 s and 4000 rpm for 45 s. Then chlorobenzene (600 μL; Alfa Aesar, 99%) was dripped as anti-solvent after 25 s the second stage to obtain a light-brown smooth film. Afterward, the perovskite film was annealed at 100 °C for 10 min to convert to a dark-brown film. Subsequently, PCBM (15 mg dissolved in 1 mL chlorobenzene) was deposited on the cooled perovskites substrates by spin coating at 1500 rpm for 45 s, followed by the spin coating of BCP saturated solution in isopropanol. Finally, silver electrode (70 nm thick) was thermally evaporated on top of the device under high vacuum (<1 × 10^−4^ Pa). The active area of the device was 0.090 cm^2^, defined by the aperture area of the metal shadow mask.

### Characterizations

X-ray diffraction (XRD) patterns were obtained with Smart LAB instruments CuKα beam (*λ* = 1.54 Å). UV-vis absorption spectra measurement was carried out on a Hitachi U-3010 spectroscope, and was employed to assess the absorption properties of the doped perovskite sensitized NiO thin film. The morphology of the film was tested with scanning electron microscopy (SEM; JEOL JSM-7401F). The incident photon-to-electron conversion efficiency (IPCE) spectra were measured in air with equipment developed by the Institute of Physics, Chinese Academy of Sciences.

The energy dispersive X-ray spectroscope (EDS) combined with a field-emission scanning electron microscope (SEM-EDS, EDAX Octane Pro). X-ray energies corresponded to I, Pb and Eu were collected as the SEM scanned the electron beam over the surface and cross-sectional area in FTO substrate. The X-ray data was synchronized with the SEM image and an ‘element image’ was created showing the presence of the selected element throughout the selected area.

The current density–voltage ( *J*–*V*) curves were measured with a 2400 Series SourceMeter (Keithley Instruments) under simulated AM 1.5 sunlight at an equivalent to 100 mW cm^−2^ irradiance generated by an thermo oriel 91192-1000 simulator, with the intensity being calibrated with an VLSI standards incorporated PN 91150V Si reference cell. The mismatch factor was calculated to be less than 1%. The solar cells were masked with a metal aperture to define the active area, typically 0.090 cm^2^. The backward bias for stability characterization of the solar cell was held to 0.75 V. The as-prepared solar cells were stored at 25 °C in light with a relative humidity (RH) of 30 ± 5% for the characterization of ambience stability. The specific PCE as a function of time was obtained with conventional environment treatment for 13 days in order to clarify the PCE evolution of solar cells.

## Results and discussion

**Fig. 1:**
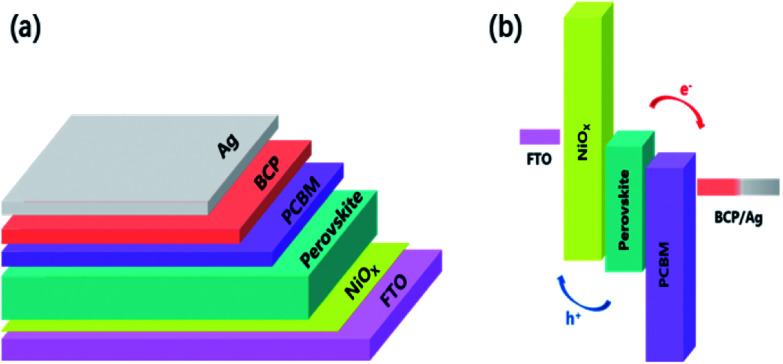
(a) The schematic architecture of the perovskite solar cells (PSCs). (b) Energy-level diagram of the PSCs, exhibiting the collecting process of photo-generated carriers.

The simple schematic diagram of our working is showed in Fig. 1 The schematic architecture of the perovskite solar cells (PSCs) and the energy band diagram of the PSCs, exhibit the collecting process of photo-generated carriers, which specific numerical referenced those reported.^56,57^

**Fig. 2:**
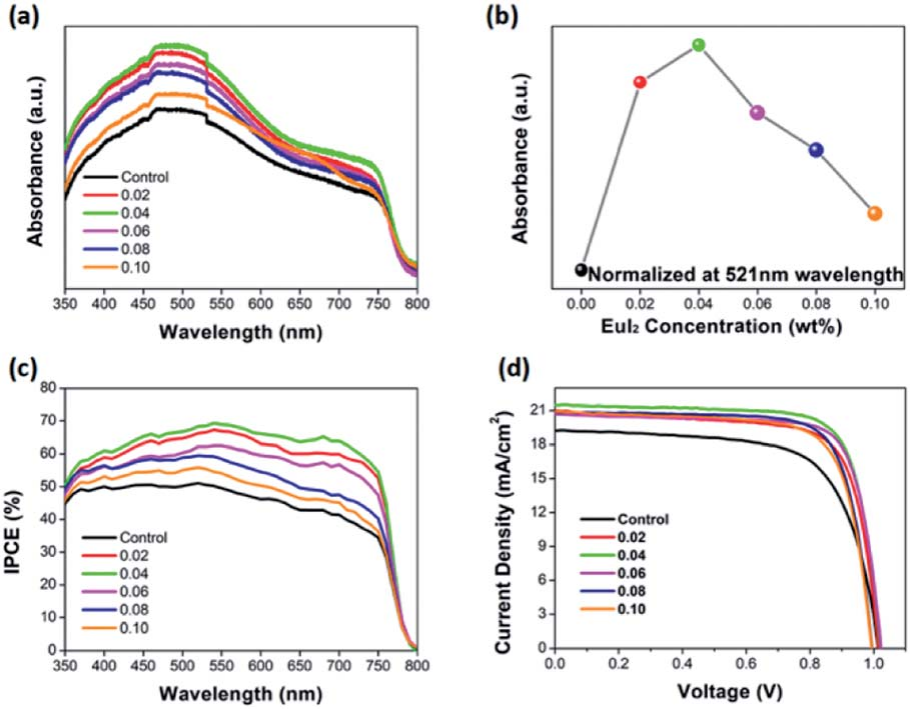
(a) UV-vis absorption spectra of the pristine and Eu^2+^-doped perovskite films. (b) The normalized absorbance at 521 nm. (c) The IPCE spectra. (d) *J*–*V* curves of the best-performing device without and with EuI_2_ doped at a scan rate of 0.10 s^−1^ under simulated sunlight irradiance.

Fig. 2 shows the absorbance result of the perovskite film with or without Eu^2+^ doped. Fig. 2(a) demonstrates the evolution of the UV-vis absorbance of perovskite films with or without doped of different EuI_2_ concentrations. Compared with the control sample, the absorbance of doped perovskite is enhanced especially in the 350–540 nm. The normalized absorbance at 521 nm is listed with different amount of EuI_2_ as the dopant in the MAPbI_3_, as shown in Fig. 2(b). At the doping concentration of 0.06%, the film exhibits the maximum absorbance. However, for the wavelength larger than 540 nm, only the perovskite film with 0.04% doped in MAPbI_3_ has an obvious improvement. As the result of our measurement, the concentration of 0.04% being doped in MAPbI_3_ achieves the highest efficiency. This probably because the rare earth Eu^2+^ in MAPbI_3_ can form a new impurity energy level, causing the absorbance spectra shift to lower energy region.^58^ It is commonly known that substances always tend to transfer to lower energy states, since the lower energy level is, the more stable substances will be.^59^ Thereby the erosion of the perovskite is slightly shifted as well as the similar phenomenon observed in de Quilettes D. W. work.^48^ As presented in Fig. 2(c), the IPCE values are significantly enhanced by doping Eu^2+^. Fig. 2(d) gives the *J*–*V* curves of the perovskite solar cells measured at backward scan direction, and the corresponding photovoltaic performance parameters are listed in Table 1, which consists with the improvement of UV-vis absorption and IPCE.

**Table 1:** The key photovoltaic parameters of the perovskite solar cell with Eu^2+^ doped and pristine control perovskite device. The active area of each PSCs is defined by a metal mask as 0.09 cm^2^

Device	*J*_sc_ (mA cm^−2^)	*V*_oc_ (V)	FF	PCE (%)
Control	19.2	1.02	0.678	13.3
0.02	20.9	1.02	0.731	15.6
0.04	21.5	1.02	0.764	16.7
0.06	20.7	1.02	0.778	16.4
0.08	20.9	1.00	0.758	15.8
0.10	20.9	1.00	0.733	15.3

**Fig. 3:**
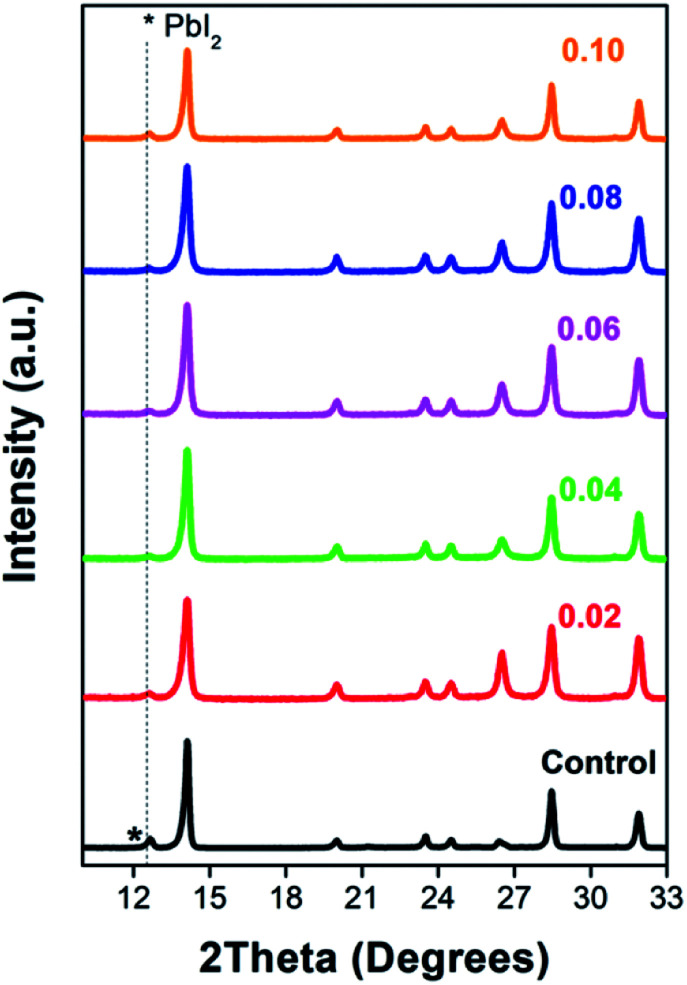
XRD patterns of the PSCs with and without EuI_2_ doped. Gray dash line depicts the position of PbI_2_.

The changes of thin film crystal structure were verified by an XRD instrument. Fig. 3 and S1† shows that both the thin films with and without EuI_2_ doping can form the typical tetragonal perovskite structure. The RE dopants do not have any significant effect on the crystallization of perovskite. Whereas the PbI_2_ diffraction peak in the XRD pattern is disappeared with EuI_2_ being doped, proving a slightly decrease of the unreacted PbI_2_. Elimination of the PbI_2_ block would effectively improve the crystal boundary contact. On one hand, the hardly existed PbI_2_ phase increase the FF and then raises the PCE of the device. On the other hand, the compact grain boundary reduces the corrosion rate of perovskite thin film and then enhances the stability of the device.

**Fig. 4:**
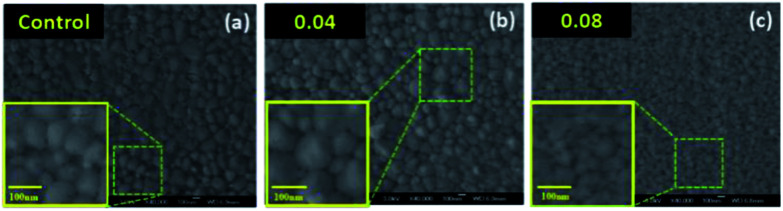
Top-down SEM images of perovskite thin films prepare by different EuI_2_ concentrations.

Scanning electron microscopy (SEM) was conducted to characterize the morphology and crystallinity of the prepared perovskite films. Fig. 4 (Fig. S2†) exhibits the top-view SEM images of the perovskite thin films prepared by adjusting the EuI_2_ concentration to tune the coverage of the film. For the control perovskite film, apparent pin-holes can be seen. It is widely known that pin-hole in the perovskite thin film will cause severe carrier recombination and deteriorate the efficiency of device. The defects not only make the electrons and holes meet directly at selective layer, but also immensely affect the absorbance.^60–62^ When the EuI_2_ as a dopant inserts into the perovskite layer, the grain size of the perovskite thin film is enlarged. When the doping concentration is 0.04 wt%, the grain size obviously larger than the pristine. Combining the XRD results, we conclude that the introducing of EuI_2_ as a dopant in the planar perovskite can improve both the film uniformity and the perovskite crystallinity, which are two key prerequisites for high performance and stable perovskite solar cells.^63^ In addition, when the doping concentration is 0.08 wt%, the grain into dendritic crystals, because crystallization rate is too fast to form independent grain.^64,65^

**Fig. 5:**
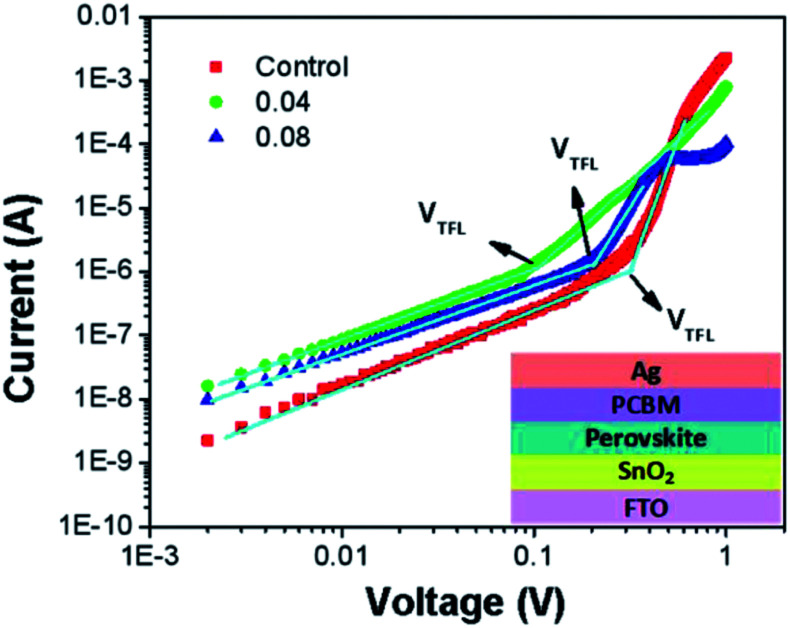
Dark *I*–*V* characteristics of the electron only device displaying *V*_TFL_ kink point behavior (inset shows the device structure).

To further investigate the electron trap-state density in the perovskite absorber, we fabricated electron-only devices (FTO/SnO_2_/MAPb_1−*x*_Eu_*x*_I_3_/PCBM/Ag). Fig. 5 shows the typical dark *J*–*V* characteristics of the electron-only and hole-only device based on different perovskites. The linear relation indicates an ohmic response of the device at low bias corresponds. The current increased linearly with the voltage up to a clear konk point. The current shows a more rapid rise, when the voltage increased to the region over the kink-point, demonstrating that filling of the trap states by injected carriers. The trap densities can be determined by the trap-filled limit voltage (*V*_TFL_) which called the space charge limited current (SCLC) technique. The trap density can be calculated by the equation:^66^*V*_TFL_ = *en*_t_*L*^2^/2 *εε*_o_, where *n*_t_ is the trap-state density, *L* is the perovskite film thickness, *ε* and *ε*_o_ represent the elementary charge and vacuum permittivity, respectively. And the relative dielectric constant of MAPbI_3_*ε* is 28.8,^67^ the thickness of measures films is 250 nm. After fitting, the *V*_TFL_ of the control, 0.04, and 0.08 are 0.3, 0.1 and 0.2 V, respectively. Therefore, the corresponding electron trap density is 8.06 × 10^15^ cm^−3^ for the control perovskite film. When the perovskite with Eu^2+^ doped at 0.04%, the electron trap density is reduced to 2.67 × 10^15^ cm^−3^, indicating that the film quality is improved. The reduced electron trap density contributed to reducing the *J*–*V* hysteresis in the perovskite with Eu doped device.

**Fig. 6:**
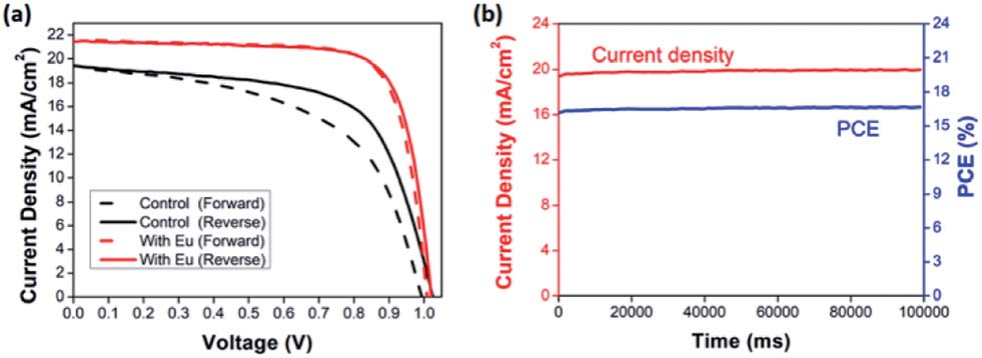
(a) *J*–*V* curves measured under both forward and reverse scan mode for the devices based on with and without Eu doping in MAPbI_3_. (b) The steady-state power and photocurrent density output as a function of time at the maximum power output values for champion device.

The hysteresis of *J*–*V* curves, which affected by the defect states or the band bending, is an important issue to determined the actual PCE. As shown in Fig. 6(a), while the devices with Eu doping, the *J*–*V* hysteresis is eliminated. That might be associated with two reasons, one is the large grain formation caused by the doping with Eu, leading to enhanced the film quality, and the other is PbI_2_ phase disappearing which reduced film defects. Table 2 summarizes the key *J*–*V* parameters of PSCs device using both reverse and forward scan conditions. In order to further confirm that there is no hysteresis indeed for the device with Eu doping and ensure the *J*–*V* measurement is reliable, the photocurrent density and PCE of the champion device were recorded as a function of time, as shown in Fig. 6(b). The photocurrent density and steady-state power output of the champion device with the doping concentration of 0.04%, remained stable within 100 s, and a highly stable PCE at 16.7% can be obtained.^68^

**Table 2:** The key *J*–*V* parameters of PSCs device using both reverse and forward scan conditions

device	Scan direction	*J*_sc_ (mA cm^−2^)	*V*_oc_ (V)	FF	PCE (%)
Control	Reverse	19.26	0.99	0.689	13.4
	Forward	19.45	1.03	0.635	12.7
0.04	Reverse	21.50	1.02	0.763	16.7
	Forward	21.56	1.01	0.762	16.4

**Fig. 7:**
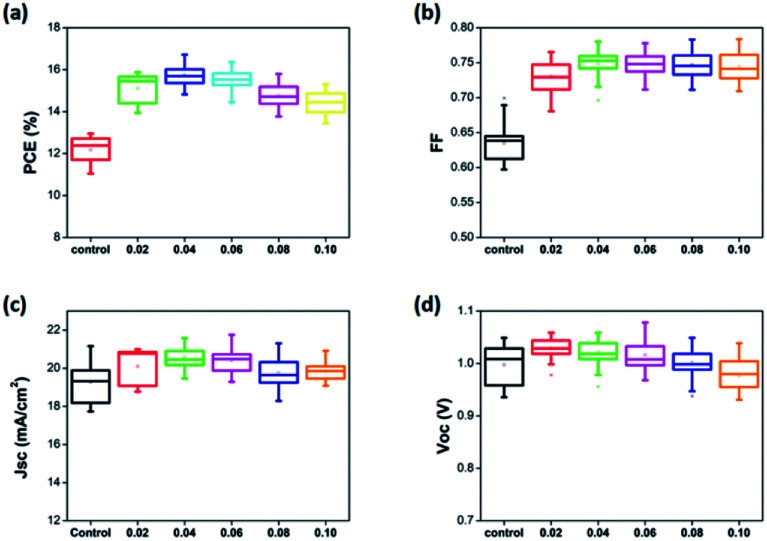
Photovoltaic characteristics for the PSCs with EuI_2_ doped of different concentrations (statistics of data for 10 batches of the device).

As mentioned above, an optimized amount of EuI_2_ is beneficial for the formation of high performance and stable perovskite thin films. The photovoltaic performance of perovskite solar cells prepared with pristine perovskite thin films and EuI_2_-doped perovskite thin films were further studied. As shown in Fig. 7, the FF and *J*_sc_ are improved obviously when EuI_2_ is applied, leading to a substantially improvement of average PCE from 13.3% to 16.7%. The statistical PCE data are obtained from 200 devices of different batches. It is demonstrated by several studies that the large grain size could achieve high performance.^69–73^ Some excellent reviews described two primary benefits of growing crystals with large grain size. One is the reduced interfacial area associated with large grains suppresses charge trapping and eliminates hysteresis. The other is larger grains have lower bulk defects and higher mobility, allowing the photo induced carriers propagate through the device without frequent encounters with defects and impurities.^71^ As can be seen from Fig. 4, when the doping concentration maintained in a certain range, the grain size has been was enlarged. While the crystals morphology becomes poor again when the EuI_2_ content reaches 0.08%, due to phase separation resulting from immiscibility, and grew into dendritic crystal.^56,70,71^

**Fig. 8:**
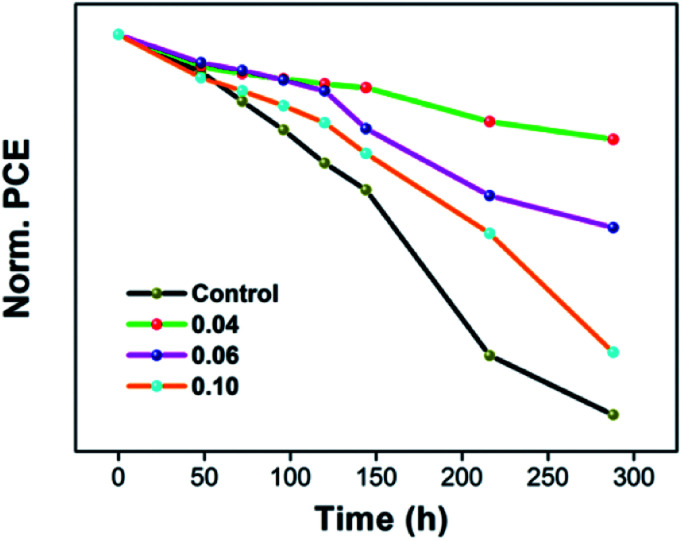
The normalized data of stabilized power output of the cells.

The normalized data of stabilized power output of the cells was shown Fig. 8. We studied the conventional environment stability of the devices, and the PCEs were measured every day. The aging test was carried out at the temperature of 25 °C and RH of 25 ± 5%. For the pristine devices, the PCE decrease nearly 48.05% in 288 h. With 0.04% of EuI_2_ doped, the top PCE only reduce by 10.25%, and maintains almost 90%. With the increase of doping concentration, the stability of the device can be improved. But if the doping concentration is too large, the stability begin to decline. As our previous analysis, the doped crystal hardly has any PbI_2_ phase precipitates, which can improve the thin film uniformity and the perovskite crystallinity. And the crystal of enlarged grain denser, make the grain boundary have less flaws, hence obtaining a high quality crystalline. High quality crystalline states not only can improve the PCE and FF, but also promote the stability of device. As we all know, seed crystals can speed up the crystallization and induce the grain boundary with less defects, which can reduce the rate of corrosion. Therefore, so our work results show the consequence that the stability of solar cells is enhanced. Furthermore, there was a positive correlation between the grain boundary area and corrosion rate, since the larger area is, the faster corrosion rate.^74,75^ For the crystal growing into dendrite, the grain boundary area is too large, thus the stability is lower, which is keeping with our test results.

**Fig. 9:**
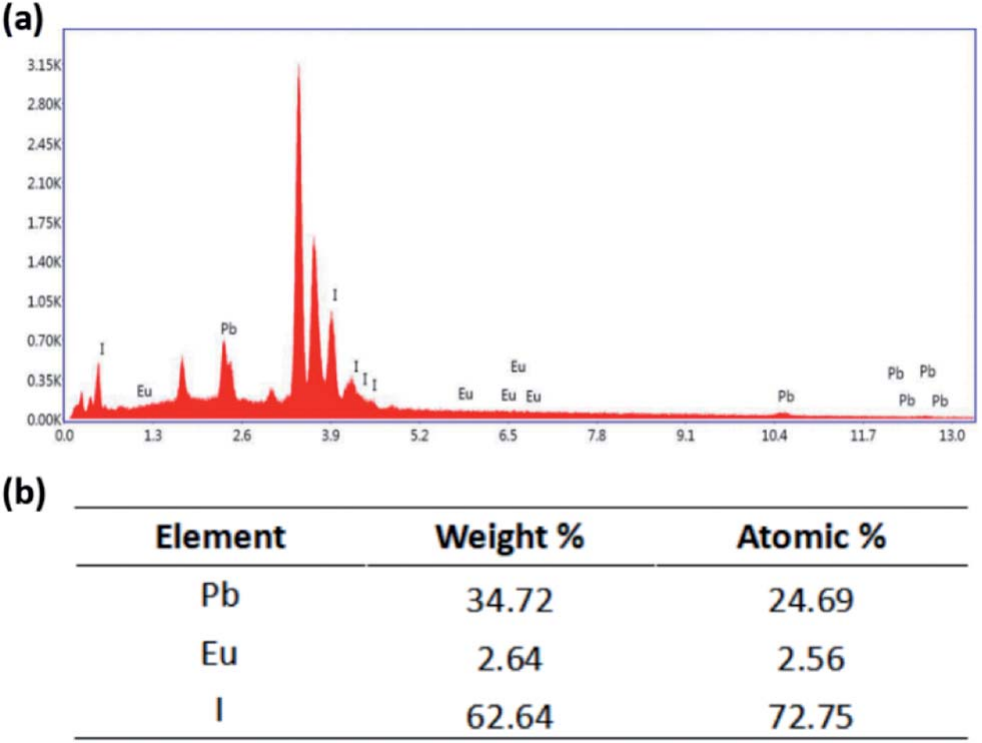
EDS results of the perovskite film on FTO substrate.

In order to characterize the doping of Eu^2+^, the SEM-EDS measurement was conducted as shown in Fig. 9. It shows the surface EDS mapping (Fig. S3†), where Pb and I are well distributed three-dimensionally in the FTO film. As shown in Fig. 7, weight percentages from EDS elemental analysis of the film surface are found to be 34.72%, 2.64% and 62.64% for Pb, Eu, and I, respectively, which correspond to 24.69%, 2.56% and 72.75% in atomic percentage. This indicates that the ratio of Pb and Eu to I is 1 : 3, which consists with the atomic ratio in the ABX_3_ structure.

## Conclusions

In conclusion, we inserted an anhydrous EuI_2_ as a dopant into the perovskite to control the perovskite crystal grain size and reduce the PbI_2_ phase of the perovskite thin film. Inserting RE elements of Eu into MAPbI_3_ is favorable to realize of a dense perovskite film with a nearly whole coverage and larger grain size. We successfully used this method to improve the FF of the inverted planar perovskite solar cells. The best performing device, using an extremely low doping ratio 0f 0.04 EuI_2_ as the dopant in MAPbI_3_, achieves a PCE of 16.7% with a high *J*_sc_ of 21.5 mA cm^−2^ and an enhanced FF of 0.763, which is much higher than the control device without any dopants. Meanwhile, EuI_2_ as a dopant greatly enhances the stability of the perovskite films. This study provides significant development towards the critical role of the RE as dopants utilizing in photovoltaic devices, and helps to boost the performance of the inverted planar perovskite solar cells to be a higher efficient and more stable state.

## Conflicts of interest

There are no conflicts to declare.

## Supplementary Material

RA-008-C7RA12754E-s001
