# Supplementary material for: CH3NH3Pb1−xEuxI3 mixed halide perovskite for hybrid solar cells: the impact of divalent europium doping on efficiency and stability
Source: RSC Adv. 2018 Mar 20;8(20):11095–101. doi: 10.1039/c7ra12754e (PMC9078983; doi:10.1039/c7ra12754e)
Supplement: RA-008-C7RA12754E-s001 [file RA-008-C7RA12754E-s001.pdf]

Electronic Supplementary Material (ESI) for RSC Advances.
This journal is © The Royal Society of Chemistry 2018

Supporting Information

$CH_3NH_3Pb_{1-x}Eu_xI_3$ mixed halide perovskite for hybrid solar cells：the impact of divalent Europium doping on the efficiency and stability

Xiaowei Wu[a], Hongwei Li[a*], Kai Wang[b], Xiaowei Sun[b] and Liduo Wang[c*]

[a] *National Engineering Research Center for Rare Earth Materials, General Research Institute for Nonferrous Metals, Grirem Advanced Materials Co., Ltd., Beijing, 100088,China. E-mail: lihw0923@vip.126.com*

[b] *Department of Electrical & Electronic Engineering, Southern University of Science and Technology of China, Shenzhen, 518055, China.*

[c] *Key Lab of Organic Optoelectronics and Molecular Engineering of Ministry of Education, Department of Chemistry, Tsinghua University, Beijing 100084, China. E-mail: chldwang@mail.tsinghua.edu.cn*

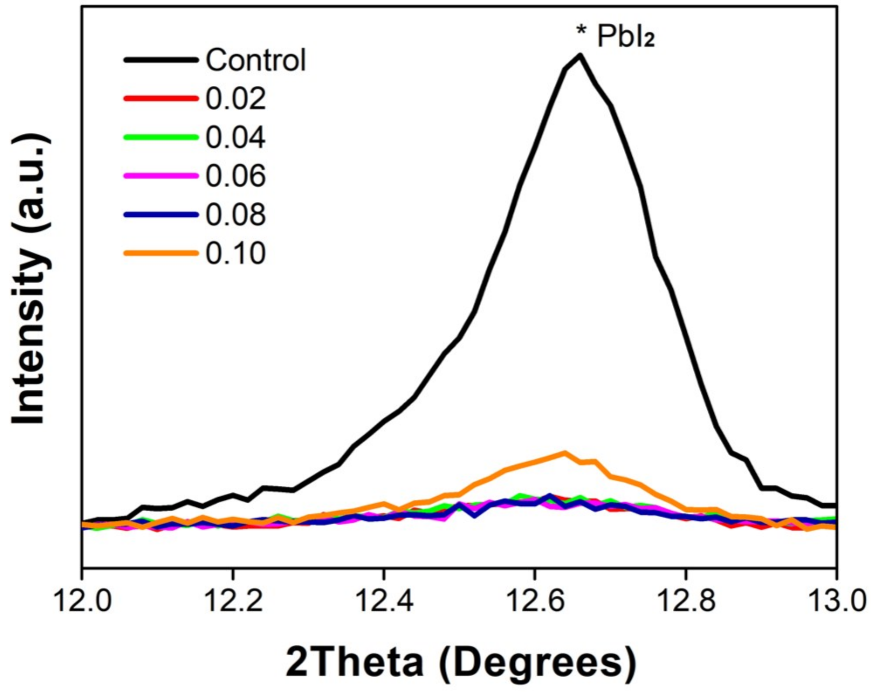

Figure S1. XRD patterns of the PSCs with and without EuI_2 doped.

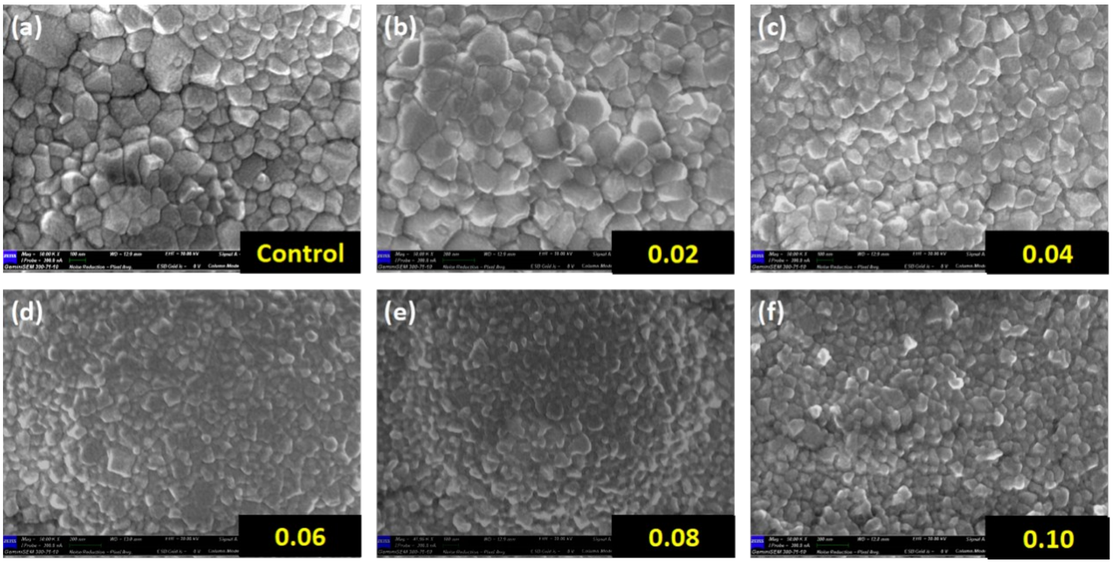

Figure S2. Top-down SEM images of perovskite thin films prepare by different EuI_2 concentrations.

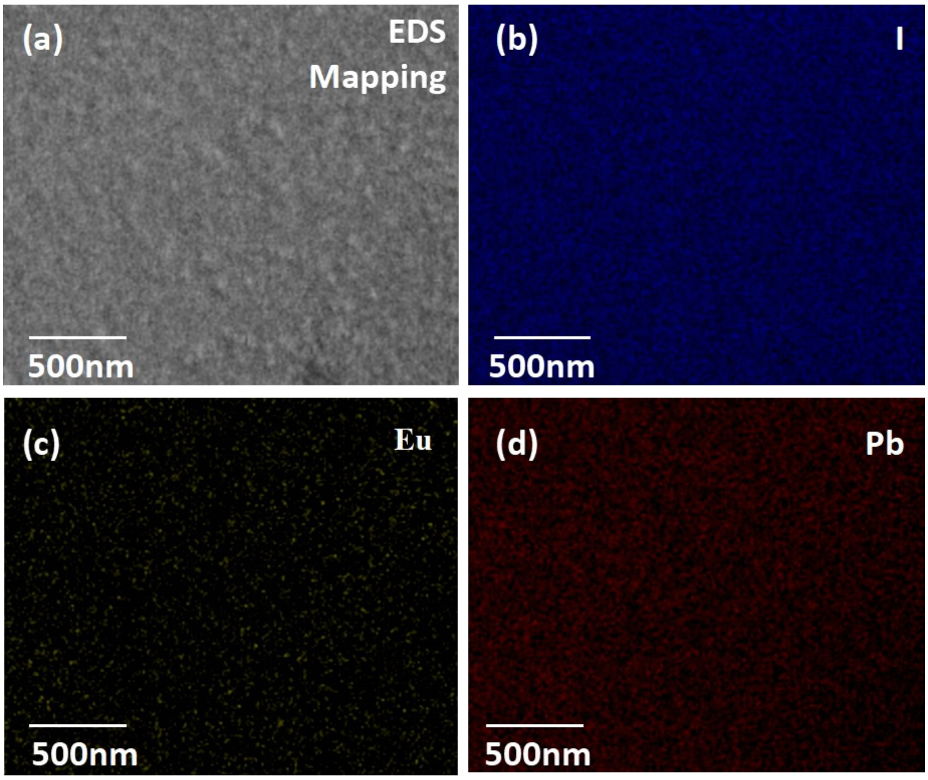

Figure S3 (a) Plan view of SEM micrographs of $CH_3NH_3Pb_{1-x}Eu_xI_3$ deposited on FTO and EDS elemental maps of (b) iodine, (c) Eu, (d) lead in the 0.10% concentration Eu^{2+}-doped perovskite films.
